# The Effect of Sub-Lethal Successive Applications of Photodynamic Therapy on *Candida albicans* Biofilm Depends on the Photosensitizer

**DOI:** 10.3390/jof9010111

**Published:** 2023-01-13

**Authors:** Luana Mendonça Dias, Marlise Inêz Klein, Túlio Morandin Ferrisse, Karine Sousa Medeiros, Cláudia Carolina Jordão, Amanda Bellini, Ana Claudia Pavarina

**Affiliations:** Department of Dental Materials and Prosthodontics, School of Dentistry, São Paulo State University (UNESP), Araraquara 14801-385, Brazil

**Keywords:** *Candida albicans*, photodynamic therapy, fluconazole

## Abstract

This study aimed to evaluate the potential of successive applications of sub-lethal doses of the antimicrobial photodynamic therapy (aPDT) mediated by Photodithazine^®^ (PDZ) and curcumin (CUR) associated with LED in the viability, reactive oxygen species (ROS) production, and gene expression of *Candida albicans*. The microbial assays were performed using planktonic cultures and biofilms. Ten successive applications (Apl#) were performed: aPDT (P+L+; C+L+), photosensitizer (P+L−; C+L−), and LED (P−L+; C−L+). Control groups were used (P−L−; C−L−). The viability of *C. albicans* was determined by cultivating treated cultures on agar plates with or without fluconazole (FLU). In addition, the ROS detection and expression of *SOD1*, *CAP1,* and *ERG11* genes were determined. For planktonic cultures, no viable colonies were observed after Apl#3 (without FLU) and Apl#2 (with FLU) for either photosensitizer. Biofilm treated with P+L+ resulted in the absence of cell viability after Apl#7, while C+L+ showed ~1.40 log_10_ increase in cell viability after Apl#2, regardless of FLU. For both photosensitizers, after the last application with viable colonies, the production of ROS was higher in the biofilms than in the planktonic cultures, and *SOD1* expression was the highest in P+L+. A reduction of *CAP1* and *ERG11* expression occurred after P+L+, regardless of FLU. C+L+ had a higher level of ROS, and the treatments were non-significant for gene expression. Sub-lethal doses of aPDT mediated by CUR could induce *C. albicans* resistance in biofilms, while *C. albicans* cells in biofilms were susceptible to aPDT mediated by PDZ.

## 1. Introduction

*Candida albicans* is a fungus present in the genitourinary and intestinal tracts, normal skin microbiota, and mucous membranes [1]. The host immunosuppression can enable this microorganism to act as an opportunistic pathogen and trigger an infection in the host [2]. The treatment approach most often used to control infections caused by *Candida* is administrating conventional antifungals [2]. However, these drugs have a limited indication due to the resistance that microorganisms can present after exposure to the drug [2]. 

On account of difficulties related to fungal resistance, antimicrobial photodynamic therapy (aPDT) has been studied and improved to promote greater safety in the process of microbial infection control [3,4,5,6,7]. The aPDT requires a photosensitizer (PS), which can be of synthetic or natural origin, a light source that must correspond to the PS absorption band, and oxygen [8]. Photodithazine^®^ (PDZ) is a commercial water-soluble glucosamine salt of chlorine(e6) [9]. Curcumin (CUR) is a natural compound with increased antifungal potential when associated with LED [4,5,10]. PDZ and CUR have shown promising results in the inactivation of *C. albicans* [4,5,7,10,11]. Microorganisms that grow as biofilm are less susceptible to aPDT than their planktonic form [4,6,7]. The organization of fungal cells in biofilm makes these cells less susceptible to antifungal drugs and aPDT [3,4,5,6,12,13].

The investigation of antimicrobial resistance and tolerance against conventional antimicrobials consists of successive exposure of the microorganism to sub-lethal (or sub-optimal) concentrations [14,15]. Sub-lethal doses of aPDT promote oxidative stress without causing irreversible changes in microbial cells, and, therefore, it is highly recommended for studies that evaluate antimicrobial resistance, tolerance, and persistence against aPDT applications [15]. In this context, the potential of sub-lethal doses of aPDT on some microorganisms has been investigated [7,16,17,18,19]. However, there is a lack of methodology applied regarding the ability of a microorganism to develop susceptibility, tolerance, or resistance to aPDT. Thus, the present study aimed to evaluate the effect of 10 consecutive applications of sub-lethal doses of aPDT mediated by PDZ and CUR in microbial viability, ROS production, and gene expression (i.e., *SOD1*, *CAP1,* and *ERG11*) against *C. albicans* cultivated in planktonic cultures and biofilms. The fungal viability of the treated cultures was assessed on culture media with and without fluconazole.

## 2. Materials and Methods

### 2.1. Preparation of PS and Irradiation Conditions

Photodithazine^®^ (PDZ, Russia, VETAGRAND, Co. Moscow, Russia) at 500 mg/mL (stock solution) was diluted in each experiment using phosphate-buffered saline (PBS, pH 7.2) to achieve a 25 mg/L working solution [6]. A red LED (Biotable 3.4, São Carlos, Brazil) emitter was used at a wavelength of 660 nm to excite the PS. The sub-lethal LED dose evaluated was 18 J/cm^2^, with 20 min pre-irradiation and 9 min of exposure to LED [6]. 

A solution of 368.38 µM curcumin (Sigma Aldrich, St. Louis, MO, USA) (>65% pure) was prepared in 2.5% dimethylsulfoxide (Quimica, São Paulo, Brazil) and diluted in PBS solution to obtain a 40 µM working solution. The samples were illuminated with an LED light (Biotable 3.4, São Carlos, Brazil) fixture in the blue region of the spectrum (450 nm), with a constant output power of 47 mW/cm^2^, at a dose of light also sub-lethal of 18 J/cm^2^. The pre-irradiation time was 20 min, followed by lighting for 6.38 min [5].

### 2.2. C. albicans Cultures and Treatments

#### 2.2.1. Growth of the Microorganism in Planktonic Culture

The standard strain of *C. albicans* ATCC 90028 (stored at −80 °C) was thawed and plated on a Petri dish with SDA culture medium (Sabouraud dextrose agar) supplemented with chloramphenicol (50 mg/L) and incubated aerobically at 37 °C for 48 h. For the starter cultures, five colonies were inoculated in 10 mL of yeast nitrogen base (YNB) at pH 7.0 supplemented with 100 mM of glucose, and the tubes were incubated for 16 h at 37 °C. Then, the starter culture was diluted in fresh medium YNB and incubated (37 °C) until the new culture reached the middle of the exponential growth phase (optical density or OD5_40nm_: 0.55 ± 0.08). This culture was adjusted to 10^7^ CFU/mL by washing the cells via centrifugation (4000× *g* for 5 min) and rinsing with PBS solution. These procedures were repeated three times. 

**Figure 1:**
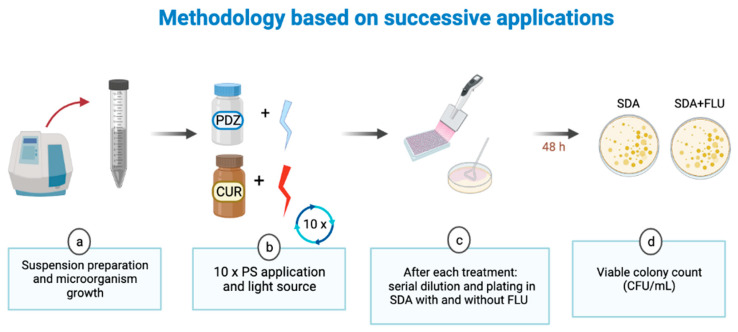
Flowchart of methodology based on successive applications of aPDT used in the present study.

Aliquots of 100 μL of the fungal cells were distributed in 96-well microplates for the treatments. Additionally, 100 μL of the PS solution was inserted into these same wells. After pre-irradiation time (20 min) the ilumination was performed (18 J/cm^2^). Then, aliquots of microrganism from each well were subjected to serial dilutions in PBS and plated on SDA supplemented with fluconazole in duplicate. The plates were then aerobically incubated at 37 °C for 48 h. After incubation, yeast colony counts of each plate were quantified using a digital colony counter (CP 600 Plus, Phoenix Ind Com Equipamentos Científicos Ltda, Araraquara, SP, Brazil) and colony forming unit per milliliter (CFU/mL) was determined. Ten successive applications (Apl#1, Apl#2, Apl#3, Apl#4, Apl#5, Apl#6, Apl#7, Apl#8, Apl#9, and Apl#10) of the treatments were performed, followed by culture dilution and plating (Figure 1). The experimental groups constituted were: P+L+ (PDZ + red LED) (aPDT); C+L+ (CUR + blue LED) (aPDT); P+L− (only PDZ); C+L− (only CUR); P−L+ (red LED); C−L+ (blue LED); and P−L− and C−L− (experimental control/absence of PS and light application). The experiments were carried out in quadruplicate on three different occasions (*n* = 12).

#### 2.2.2. *C. albicans* Biofilm Formation and Treatments

For biofilm formation, 100 µL of the culture adjusted to 10^7^ CFU/mL (YNB) and 100 µL of YNB medium were plated in 96-well microplates and incubated at 37 °C for 1.5 h (orbital shaking at 75 rpm) to achieve the adhesion phase. Next, the wells were washed 3X with PBS to remove the cells not adhered, and then 200 µL of RPMI 1640 (Roswell Park Memorial Institute) buffered with 3-(N-morpholino) propanesulfonic acid (MOPS) (pH = 7.2) was inserted into the same wells that contained the adhered cells. The plates were incubated at 37 °C for 48 h. When biofilm formation reached 24 h, the medium was changed via aspiration and the addition of the same fresh medium. At 48 h of biofilm formation, the treatments were carried out in the same experimental groups described previously (Section 2.2.1—*C. albicans* cultures and treatments/growth of the microorganism in planktonic). Then, each well was scraped for 45 s [7]. The experiments were performed in quadruplicate on three different occasions (*n* = 12).

### 2.3. Cell Viability Analysis (CFU/mL)

After the treatments and the releasing of the biofilms from each well, aliquots of 100 µL of samples from each group evaluated were subjected to serial dilution (from 10^−1^ to 10^−3^) in PBS, followed by plating on plates comprising SDA and SDA supplemented with fluconazole (sub-mic 8.0 µg/mL; SDA + FLU) [7,20]. All plates were incubated aerobically at 37 °C for 48 h. After incubation, the colonies were counted, and the number of colony-forming units per milliliter was determined (CFU/mL). The surviving cells (i.e., colonies grown on agar plates) from each application were collected and stored in microtubes containing 300 µL of RNA*later*^TM^ (Ambion) and frozen at −80 °C.

### 2.4. Quantification of ROS Production after aPDT

ROS production was quantified after the first application and in the last application when the presence of viable cells was observed. New planktonic cultures and biofilms were prepared as described in Section 2.2.1 and Section 2.2.2. After the selected treatment applications, the fluorescent probe 2′,7′-dichlorodihydrofluorescein diacetate (DCFH) (Sigma-Aldrich, St. Louis, MO, USA) was used and adjusted to a final concentration of 50 mM [11]. In addition to the experimental groups described in Section 2.2.1 (*C. albicans* cultures and treatments/growth of the microorganism in planktonic), two control groups were added: one had *C. albicans* cells adjusted to 10^7^ CFU/mL without treatment (NC), and the other group was exposed to hydrogen peroxide (10 mM H_2_O_2_) (Sigma-Aldrich, St. Louis, MO, USA) [11]. Initially, the volume of 50 μL of the inoculum and 50 μL of the solutions containing the PS (PDZ and CUR) were inserted into the microtubes and centrifuged (at 4000× *g* for 5 min). The samples were adjusted (10^7^ CFU/mL), and a volume of 100 μL and then 70 μL of the fluorophore solution previously prepared was added. The microtubes were wrapped in aluminum foil and kept for one hour on the orbital shaker (30 °C, 120 rpm). Afterward, the samples were subjected to LED (P+L+, P−L+, C+L+, C−L+) at a dose of 18 J/cm^2^. The groups P+L− and P−L− or C+L− and C−L− remained incubated in the dark during lighting. In addition, the groups H_2_O_2_ and NC were maintained for 30 min in orbital agitation (30 °C, 120 rpm) during lighting. All groups were transferred to the same black well plate with 96 wells without surface treatment. Finally, the plate was read on the Fluorskan Ascent device (Thermo Scientific, Waltham, MA, USA), using the Ascent Software 2.0 program, in the spectral range of excitation of 485 nm and emission of 538 nm. The experiments were carried out in quadruplicate on three different occasions (*n* = 12).

### 2.5. RT-qPCR Analysis

The RT-qPCR test was applied to the surviving *C. albicans* cells recovered from biofilms after Apl#1, #4, and #7 of aPDT mediated by PDZ (growing on plates SDA and SDA + FLU) and Apl#1, #3, #6, and #10 of aPDT mediated by CUR (growing on plates SDA and SDA + FLU). These applications were chosen due to the results found in the microbial viability load showing a progressive reduction (aPDT mediated by PDZ) and an increase (aPDT mediated by CUR) of *C. albicans* over the ten applications. 

The phenol–chloroform with ethanol precipitation method was used for RNA extraction [21] with a mechanical cell rupture process using glass beads [22,23,24,25]. The pellet resulting from the rupture cycles was resuspended in 100 μL of H_2_O and kept on ice for 1 h for hydration. Then, RNA purification was performed with DNase on column (RNeasy Micro Kit, Qiagen, Hilden, Germany) and solution (Turbo DNase; Ambion, Austin, TX, USA) [21]. Agarose gel was used to analyze the RNA integrity (1% agarose containing 0.3 μg/mL ethidium bromide). The images were acquired using a gel-doc system (Bio-Rad, Laboratories, Hercules, CA, USA). The RNA yield and purity were verified using a nanospectrophotometer (OD_260nm_ e OD_260/280ratio_) (DS-11, DeNovix, Wilmington, DE, USA).

For cDNA synthesis, 1 μg of RNA was used for reverse transcription with the iScript cDNA Synthesis kit (Bio-Rad Laboratories, Alfred Nobel Drive, Hercules, CA, USA), following the manufacturer’s instructions. Negative controls were prepared with all reagents except the reverse transcriptase to ensure the absence of DNA contamination and consequent amplification. After the procedures, all samples were stored at −20 °C. RT-qPCR analysis was performed for *SOD1* (oxidative stress), *CAP1* (oxidative stress and virulence), and *ERG11* (ergosterol synthesis) genes (Table 1). 

The standard curves for the qPCR reactions were prepared by conventional PCR using the Taq DNA Polymerase, recombinant kit (Life Technologies, Carlsbad, CA, USA). For this, 1 μL of genomic DNA of *C. albicans* (200 ng/μL), 1 μL of 10 μM primers (Table 1), 5 μL of iTaq Buffer 10×, 1.5 μL of MgCl_2_ (50 mM), 1 μL of dNTPs (10 mM), 40.25 μL of molecular grade water, and 0.25 μL of iTaq DNA polymerase (5 U/μL) were used for a final volume of 50 μL. A thermal cycler, CFX96 Touch (Bio-Rad Laboratories, Hercules, CA, USA), was used to run the PCR reactions, following the cycle protocol of: 3 min at 95 °C, 30 cycles of 15 s at 95 °C, 50–60 °C (adjusted per primer) for 30 s, and 72 °C for 30 s; the reactions were maintained at 4 °C. PCR products were purified (QI Aquick PCR Purification Kit, Qiagen, Hilden, Germany) according to the manufacturer’s recommendations, quantified, and diluted to obtain 2 × 10^8^ copies/µL to 2 × 10^2^ copies/µL that were used for standard qPCR curves (correlation coefficient ≅ 1; reaction efficiency of 95–105% and slope of ≅−3.3). The primers’ optimal concentrations and specificity were from a previous study [22,24,26].

Each qPCR reaction was prepared with a final volume of 25 µL with 0.5 µL of cDNA template (or standard curve point), 12.5 µL of SYBR Green Supermix (Bio-Rad Laboratories, Alfred Nobel Drive, Hercules, CA, USA), adequate volume in µL of 10 µM primer set (corresponding to the optimal concentration), and volume in µL of molecular-grade water to complete 25 µL. The reactions were cycled in the CFX96-BioRad equipment as follows: step 1 (1×) 95 °C/3 min; step 2 (35×) 95 °C/15 s, Tm (°C) of each primer/30 s, 68 °C/15 s, where data collection and analysis took place in real time; step 3 (1×) 95 °C/1 min; step 4 (1×) 55 °C/1 min; and step 5 (80×) 55 °C/1 min for the melting curve analysis.

### 2.6. Statistical Analysis

For all statistical procedures, the SPSS software (version 2.0) was used, considering a significance level of 5% (α = 0.05). The Shapiro–Wilk and Leven’s tests were used to evaluate the data’s normal distribution and homoscedastic, respectively. For fungal viability evaluation, the Kruskal–Wallis test followed by Dunn’s for multiple comparisons test were applied. For ROS production, variance analysis (one-way ANOVA) followed by Welch correction and Game–Howell’s test for multiple comparisons were used. For the analysis of gene expression, two-way ANOVA followed by mean estimation ±95% confidence interval was applied.

## 3. Results

### 3.1. Cell Viability

#### 3.1.1. Cell Viability of *Candida albicans* in the Planktonic Phase

**Figure 2:**
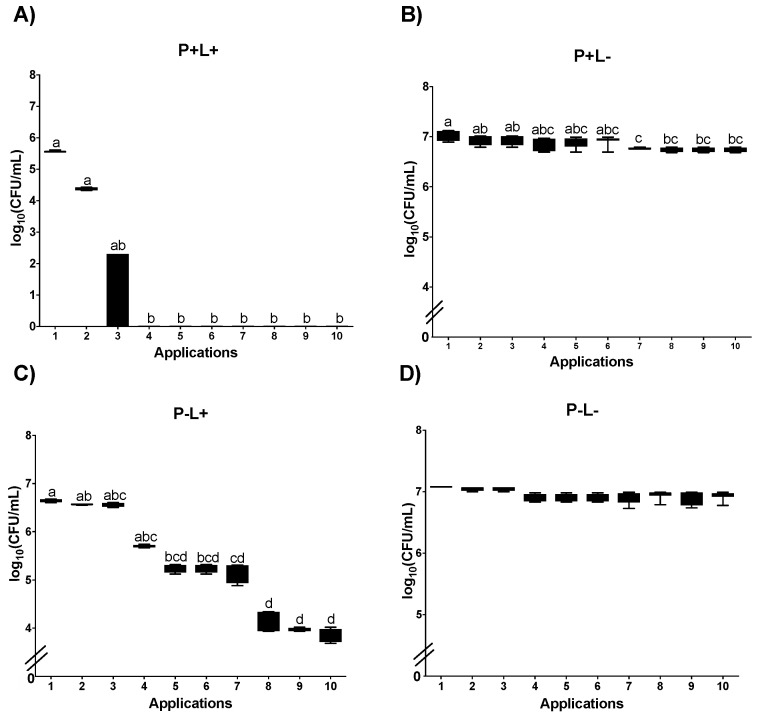
Log_10_ values (CFU/mL) of planktonic cultures of *C. albicans* submitted to (**A**) aPDT (P+L+), (**B**) PDZ (P+L−), (**C**) light (P−L+), and (**D**) control group (P−L−). Upper limit of the box: third quartile; lower limit of the box: first quartile; error bars: minimum and maximum values. Different letters (a; b; c; d) denote statistical differences between columns (applications) according to Dunn’s post-test (*p* < 0.05) (*n* = 12).

After 10 successive applications of aPDT mediated by PDZ (P+L+) in planktonic cultures of *C. albicans* and cultures plated on SDA plates, a significant difference was observed between the Apl#1 and Apl#4 with 4.5 log_10_ CFU/mL (64.2%) of microbial load reduction. In addition, after Apl#4, no growth of viable cells was observed (Figure 2A). Despite significant reductions in the planktonic cultures of *C. albicans* for the P+L− group, these were no more than 0.5 log_10_ CFU/mL (Figure 2B). For planktonic cultures submitted to light application alone (P−L+), a significant reduction was observed between Apl#1 and Apl#5 (1.4 log_10_ CFU/mL) and between Apl#1 and Apl#10 (2.8 log_10_ CFU/mL) (Figure 2C). In addition, for the P−L− group, no significant differences among the applications were observed (Figure 2D).

**Figure 3:**
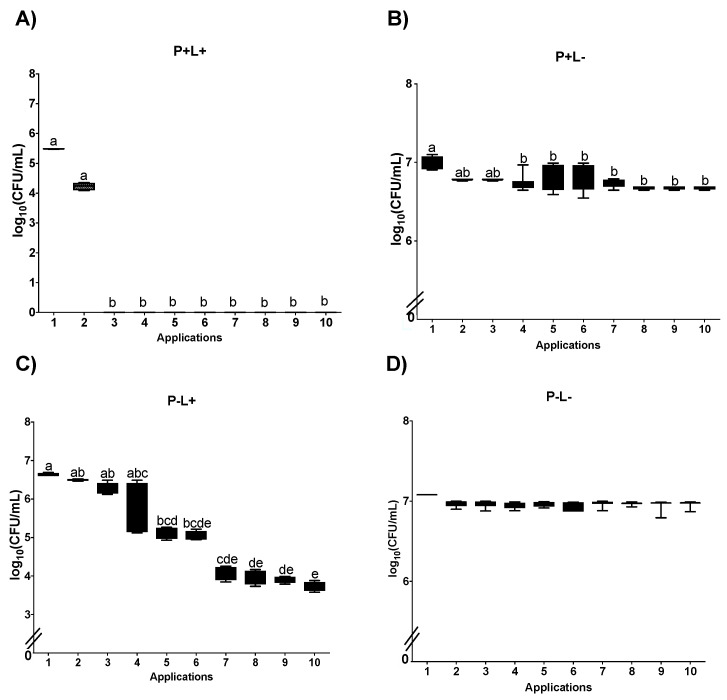
Log_10_ values (CFU/mL) of planktonic cultures of *C. albicans* submitted to aPDT (**A**) (P+L+), (**B**) PDZ (P+L−), (**C**) light (P−L+), and (**D**) experiment control (P−L−) grown in fluconazole. Upper limit of the box: third quartile; lower limit of the box: first quartile; error bars: minimum and maximum values. Different letters (a; b; c; d; e) denote statistical differences between columns (applications) according to Dunn’s post-test (*p* < 0.05) (*n* = 12).

After the applications of aPDT mediated by PDZ in *C. albicans* plated in SDA containing fluconazole, a significant difference between Apl#1 and Apl#3, with a 100% decrease in viability, was found (Figure 3A). For the group P+L−, a significant microbial load reduction between Apl#1 and Apl#4 (0.3 log_10_ CFU/mL) and between Apl#1 and Apl#10 (0.36 log_10_ CFU/mL) was noted (Figure 3B). For cultures cultivated in fluconazole submitted to light application alone (P−L+), a significant reduction in the microbial load was observed between Apl#1 and Apl#5 (1.8 log_10_ CFU/mL) and between Apl#1 and Apl#10 (2.9 log_10_ CFU/mL) (Figure 3C). For the P−L− group, no significant differences were observed (Figure 3D).

**Figure 4:**
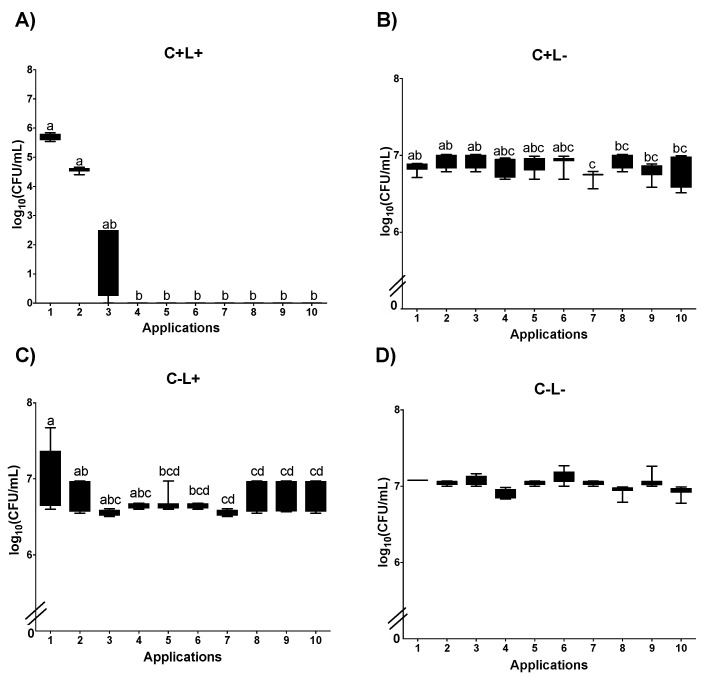
Log_10_ values (CFU/mL) of planktonic cultures of *C. albicans* submitted to (**A**) CUR (C+L+), (**B**) CUR (C+L−), (**C**) light (C−L+), and (**D**) experiment control (C−L−). Upper limit of the box: third quartile; lower limit of the box: first quartile; error bars: minimum and maximum values. Different letters (a; b; c; d) denote a statistical difference between columns (applications) according to Dunn’s post-test (*p* < 0.05) (*n* = 12).

For aPDT mediated by CUR (C+L+) in planktonic cultures plated on SDA plates, a significant reduction was noted between Apl#1 and Apl#4 (4.1 log_10_ CFU/mL (59.8%)). Furthermore, after Apl#4, no growth of viable cells was observed (Figure 4A). In the C+L− group, a significant microbial load reduction was observed between Apl#1 and Apl#7 (0.78 log_10_ CFU/mL) (Figure 4B). For cultures treated with light (C−L+), a significant reduction was observed between Apl#1 and Apl#5 (0.7 log_10_ CFU/mL) and between Apl#1 and Apl#10 (0.79 log_10_ CFU/mL) (Figure 4C). No significant difference was observed in the C−L− group (Figure 4D).

**Figure 5:**
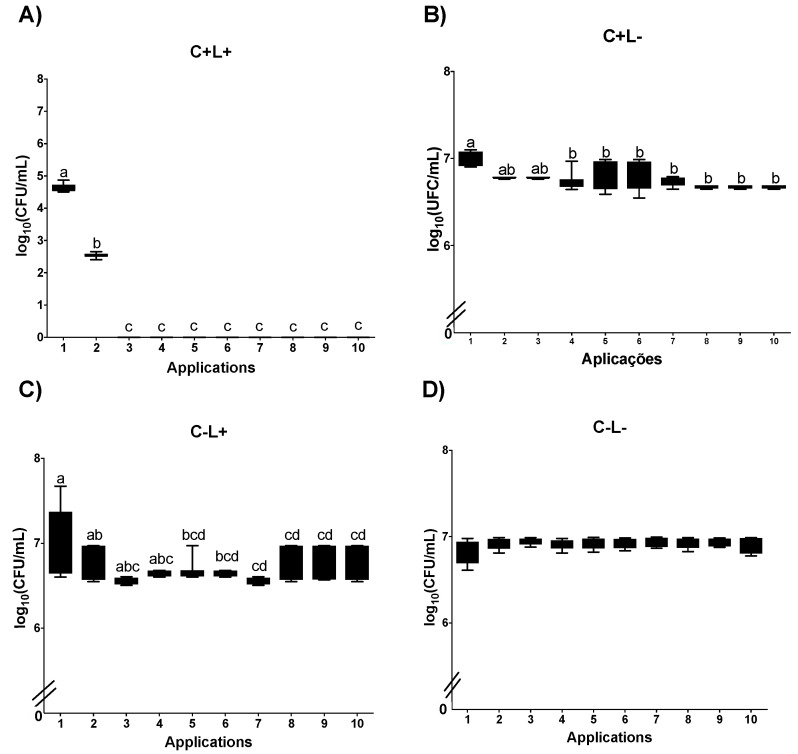
Log_10_ values (CFU/mL) of planktonic cultures of *C. albicans* submitted to (**A**) CUR (C+L+), (**B**) CUR (C+L−), (**C**) light (C−L+), and (**D**) experiment control (C−L−) grown in fluconazole. Upper limit of the box: third quartile; lower limit of the box: first quartile; error bars: minimum and maximum values. Different letters (a; b; c; d) denote a statistical difference between columns (applications) according to Dunn’s post-test (*p* < 0.05) (*n* = 12).

In curcumin-mediated aPDT (C+L+) performed in planktonic cultures of *C. albicans* cultivated on SDA containing fluconazole, a significant reduction was observed between Apl#1 and Apl#3, with a 100% decrease in viability (Figure 5A). In addition, between Apl#1 and Apl#2, a reduction of 2.09 log_10_ CFU/mL was observed, and after Apl#3, no growth of viable cells was noted (Figure 5A). No more than 1 log_10_ CFU/mL in microbial load reduction was observed for the C+L− group between Apl#1 and Apl#4 and between Apl#1 and Apl#10 (Figure 5B). For the C−L+ group, a significant difference was found between Apl#1 and Apl#5, with 0,03 log_10_ CFU/mL of reduction (Figure 5C). In addition, no significant differences were noted in the C−L− group (Figure 5D).

#### 3.1.2. Cell Viability of *C. albicans* in Biofilm

**Figure 6:**
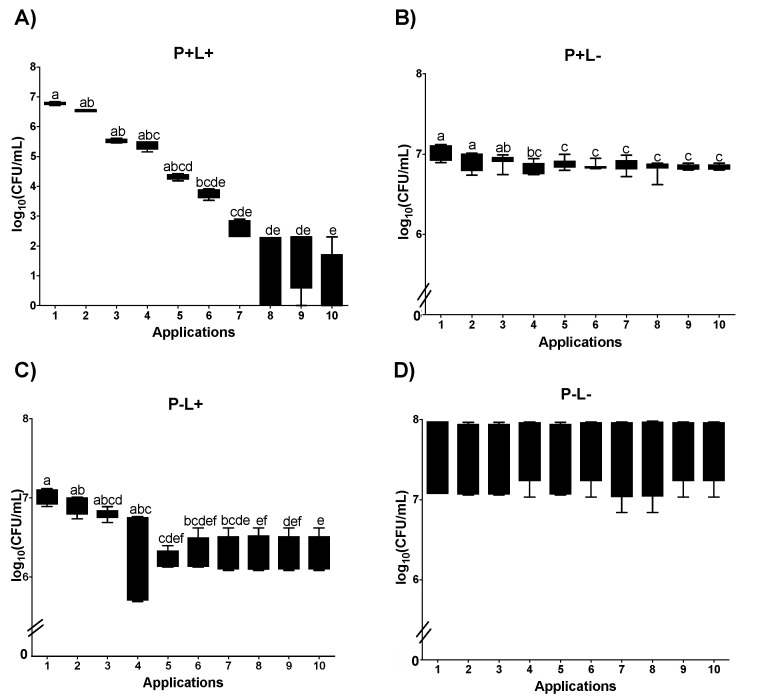
Log_10_ values (CFU/mL) of *C. albicans* biofilms submitted to (**A**) aPDT (P+L+), (**B**) PDZ (P+L−), (**C**) light (P−L+), and experiment control (**D**) (P−L−). Upper limit of the box: third quartile; lower limit of the box: first quartile; error bars: minimum and maximum values. Different letters (a; b; c; d; e; f) denote a statistical difference between columns (applications) according to Dunn’s post-test (*p* < 0.05) (*n* = 12).

For the biofilm of *C. albicans* submitted to aPDT mediated by PDZ (P+L+) plated on SDA, a significant reduction was observed between Apl#1 and Apl#6 (3.5 log_10_ CFU/mL) and between Apl#1 and Apl#10 (6.2 log_10_ CFU/mL) (Figure 6A). In the P+L− group, no more than 1 log_10_ CFU/mL of reduction in fungal viability was observed between Apl#1 and Apl#10 (Figure 6B). For the P−L+ group, a significant reduction was observed between Apl#1 and Apl#10 (0.86 log_10_ CFU/mL of drop) (Figure 6C). In addition, no significant difference was observed in the control group (P−L−) (Figure 6D).

**Figure 7:**
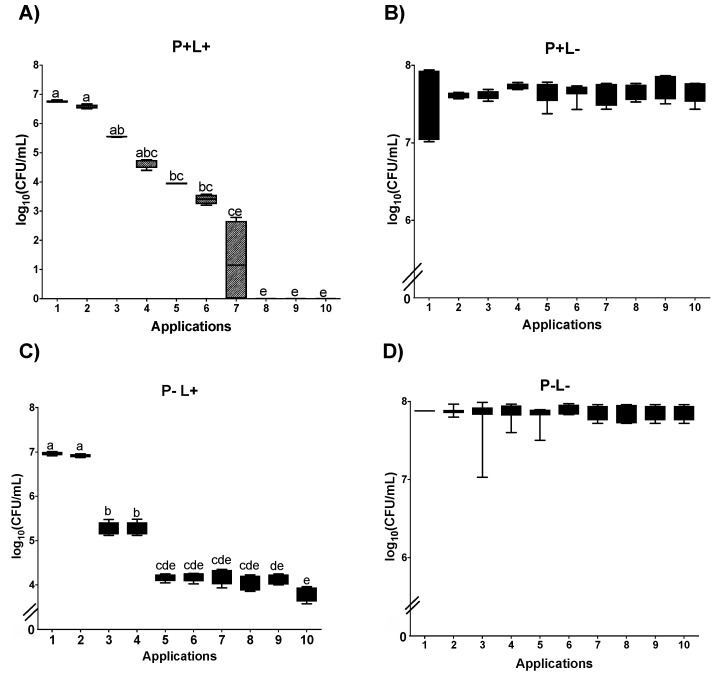
Log_10_ values (CFU/mL) of *C. albicans* biofilms submitted to (**A**) aPDT (P+L+), (**B**) PDZ (P+L−), (**C**) light (P−L+), and (**D**) experiment control (P−L−) grown in fluconazole. Upper limit of the box: third quartile; lower limit of the box: first quartile; error bars: minimum and maximum values. Different letters (a; b; c; d; e) denote statistical differences between columns (applications) according to Dunn’s post-test (*p* < 0.05) (*n* = 12).

The results of PDZ-mediated aPDT (P+L+) in *C. albicans* biofilms cultivated on SDA containing fluconazole showed a difference between Apl#1 and Apl#5, with 3 log_10_ CFU/mL of decrease in viability (Figure 7A). There were no viable cells after Apl#7. For the P−L+ group, a significant difference was observed between Apl#1 and Apl#3 and between Apl#1 and Apl#10, with a reduction in viability of 1.7 log_10_ CFU/mL and 3.1 log_10_ CFU/mL, respectively (Figure 7C). For the P+L− and P−L− groups, no statistical difference was observed among the applications (Figure 7B,D).

**Figure 8:**
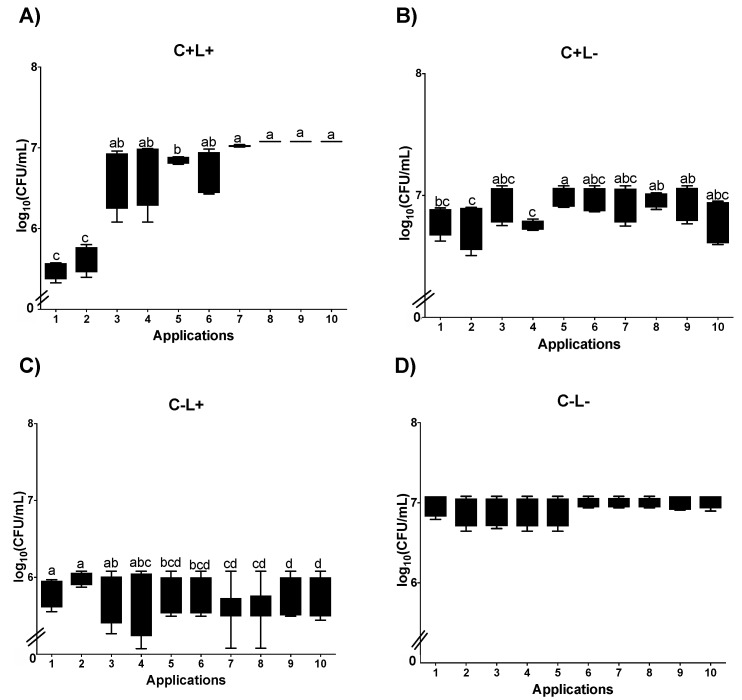
Log_10_ values (CFU/mL) of *C. albicans* biofilms submitted to (**A**) aPDT (C+L+), (**B**) CUR (C+L−), (**C**) light (C−L+), and (**D**) experiment control (C−L−). Upper limit of the box: third quartile; lower limit of the box: first quartile; error bars: minimum and maximum values. Different letters (a; b; c; d) denote statistical differences between columns (applications) according to Dunn’s post-test (*p* < 0.05) (*n* = 12).

In curcumin-mediated aPDT (C+L+), plating on SDA showed that Apl#1 promotes a reduction of ~2 log_10_ CFU/mL. The results from Apl#1 were statistically similar to Apl#2; however, after Apl#3, a significant increase in fungal viability was observed (1.39 log_10_ CFU/mL) (Figure 8A). For biofilms treated with CUR alone (C+L−), a significant difference was observed between Apl#1 and Apl#5 (Figure 8B); however, there was no difference between Apl#1 and Apl#10. For the C−L+ group, there were significant differences between Apl#1 and Apl#5, and after Apl#10, ~0.3 log_10_ CFU/mL of viability reduction was observed (Figure 8C). Furthermore, for P−L−, no significant differences were noted (Figure 8D).

**Figure 9:**
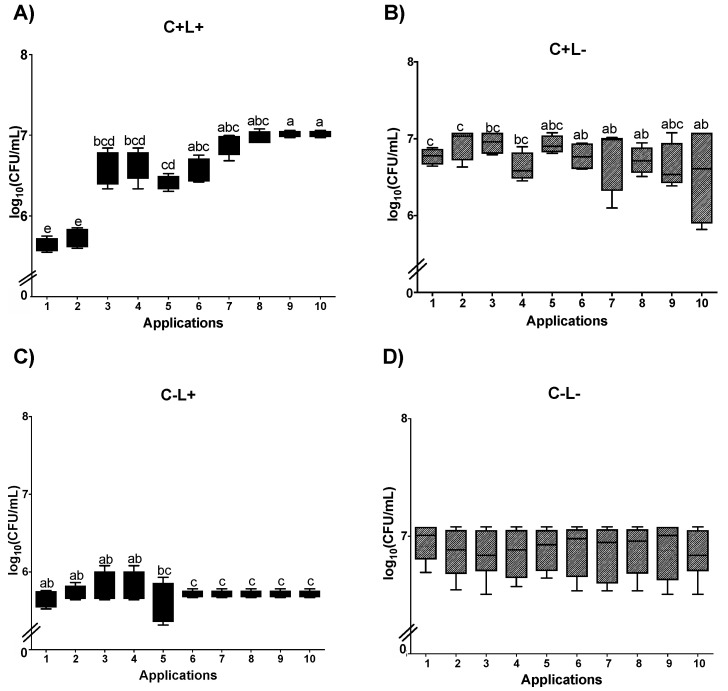
Log_10_ values (CFU/mL) of *C. albicans* biofilms submitted to (**A**) aPDT (C+L+), (**B**) CUR (C+L−), (**C**) light (C−L+), and (**D**) experiment control (C−L−) grown in fluconazole. Upper limit of the box: third quartile; lower limit of the box: first quartile; error bars: minimum and maximum values. Different letters (a; b; c; d; e) denote statistical differences between columns (applications) according to Dunn’s post-test (*p* < 0.05) (*n* = 12).

For curcumin-mediated aPDT (C+L+) in *C. albicans* biofilms cultivated in SDA with fluconazole, Apl#1 was statistically similar to Apl#2; however, after Apl#3, a significant increase in the viability of biofilm was observed (1.38 log_10_ CFU/mL of viability increases) (Figure 9A). When the biofilms were treated with curcumin alone (C+L−), a significant difference was observed between Apl#1 and Apl#6, with a reduction of 0.2 log_10_ CFU/mL (Figure 9B). For the C−L+ group, a significant difference between Apl#1 and Apl#6 with ~0.2 log_10_ of microbial reduction was observed (Figure 9C). In the P−L− group, no significant differences were noted (Figure 9D).

### 3.2. Quantification of ROS

**Figure 10:**
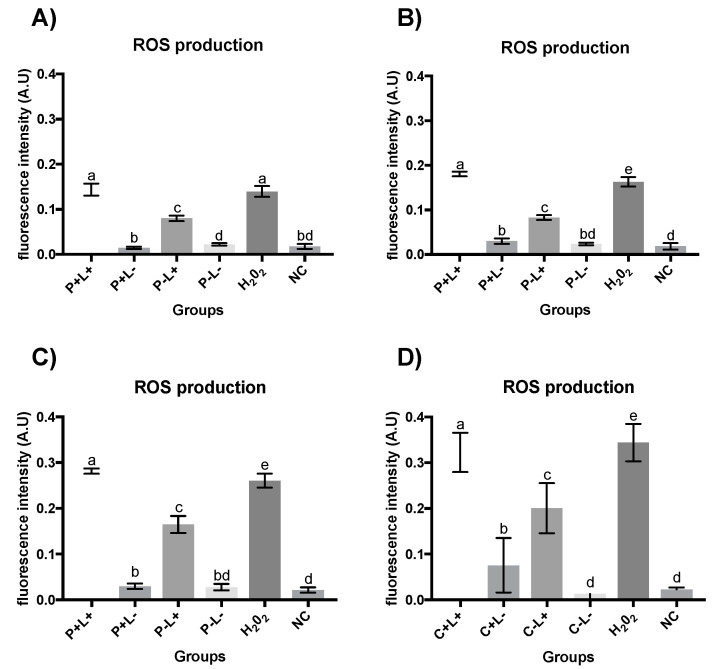
Graphic representation of the mean values and standard deviation of ROS production, measured as fluorescence intensity by fluorometric H2DCF-DA oxidation, of *C. albicans* suspensions (**A**,**B**) and biofilm (**C**,**D**) after the first application (**A**,**C**) and seventh application (**B**,**D**), subjected to aPDT (P+L+), PDZ (P+L−), light only (P−L+), no treatment (P−L−), hydrogen peroxide (H_2_O_2_), and negative control group with cells treated in no fluorochrome (NC). A.U.—arbitrary units. Different letters (a; b; c; d; e) denote statistical differences between columns (groups) according to the Games–Howell post-test (*p* < 0.05) (*n* = 12).

The intracellular production of ROS observed after Apl#1 of aPDT mediated by PDZ in planktonic cultures of *C. albicans* (Figure 10A) for the P+L+ group was similar to the H_2_O_2_ group. Furthermore, the P+L+ group differed significantly from the other groups evaluated (P−L+, P+L−, and NC group). The ROS quantification after Apl#7 of aPDT mediated by PDZ (P+L+) (Figure 10B) shows the highest levels of ROS production (average of 0.18 A.U.) being significantly different to H_2_O_2_ group (average of 0.16 A.U.). The P−L+ group presented intermediate values (mean of 0.08 A.U.), substantially different from the H_2_O_2_ group. The P+L−, P−L−, and NC groups showed a similar level of ROS production.

After Apl#1 of aPDT mediated by PDZ in biofilms of *C. albicans* (Figure 10C), the highest values of intracellular production of ROS (mean of 0.28 A.U.) were observed for the P+L+ group, with this group being significantly different to the other evaluated groups. In addition, the P−L+ group presented intermediate values (average of 0.16 A.U.). The P+L− group showed similar values to the P−L− group and to the negative control (NC). After Apl#7 of aPDT in a biofilm (Figure 10D), the P+L+ group showed similar values of ROS to the H_2_O_2_ group and significantly differed from the other groups evaluated. The P−L+ group presented intermediate and statistically different results from the other groups. Furthermore, the P−L− group was similar to the negative control (NC).

**Figure 11:**
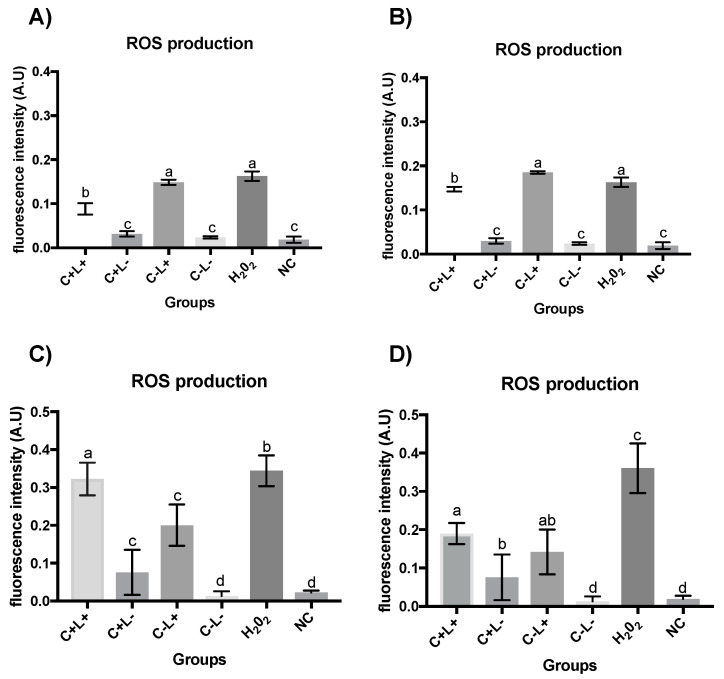
Graphic representation of the mean values and standard deviation of ROS production, measured as fluorescence intensity by fluorometric H2DCF-DA oxidation, of *C. albicans* suspensions (**A**,**B**) and biofilm (**C**,**D**) after the first application (**A**,**C**), third application (**B**), and tenth application (**D**) subjected to aPDT (C+L+), CUR (C+L−), light only (C−L+), no treatment (C−L−), hydrogen peroxide (H_2_O_2_), and negative control group with cells treated in no fluorochrome (NC). A.U.—arbitrary units. Different letters (a; b; c; d) denote statistical differences between columns (groups) according to the Games–Howell post-test (*p* < 0.05) (*n* = 12).

The ROS intracellular production performed after Apl#1 application of aPDT mediated by CUR in planktonic cultures of *C. albicans* (Figure 11A) showed that the C+L+ group was statistically different from the other experimental groups. However, the C−L+ group was statistically similar to the H_2_O_2_ group. In addition, the groups C+L−, C−L−, and NC showed the lowest levels of ROS. After Apl#3 application of aPDT (Figure 11B), the C−L+ group was similar to the H_2_O_2_ group.

After Apl#1 of aPDT mediated by CUR in biofilm (Figure 11C), the highest values of intracellular ROS (mean of 0.28 A.U.) were observed for the H_2_O_2_ group (H_2_O_2_). In addition, the C+L+ group presented intermediate values (mean of 0.20 A.U.). The C+L− group showed statistically similar values to the C−L+ group. The experimental control group (C−L−) and negative control (NC) also behaved similarly. After Apl#10 of C+L+ in biofilm (Figure 11D), similar to that observed after Apl#1, the H_2_O_2_ group showed the highest production of intracellular ROS (mean of 0.36 A.U.).

### 3.3. Results of RT-qPCR for aPDT Mediated by PDZ

**Figure 12:**
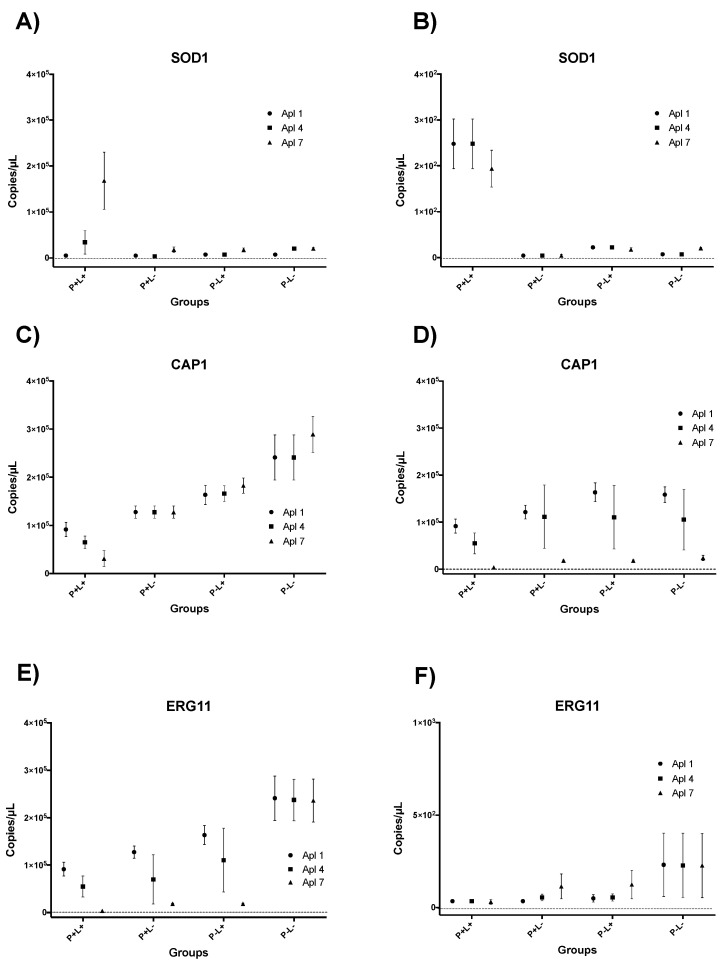
Graphic representation of mean ± confidence interval of 95% of *SOD1* (**A**,**B**), *CAP1* (**C**,**D**), and *ERG11* (**E**,**F**) gene expression in each experimental group of *C. albicans* cells removed from biofilm cultured in SDA without (**A**,**C**,**E**) and with (**B**,**D**,**F**) fluconazole after first (Apl#1), fourth (Apl#4) and seventh (Apl#7) application of PDZ-mediated aPDT (P+L+), PS control (P+L−), light control (P−L+), and experiment control group (P−L−). Minimum and maximum values of the copies/µL error bar represent the lower and upper limits of the confidence interval, respectively. The non-intersection of error bars denotes statistical difference (*p* < 0.05).

Regarding the *SOD1* expression of cells grown on SDA, the group P+L+, Apl#7 (highest means in the analysis) was statistically different between all other applications, with an increase of 1.5 × 10^5^ copies/µL compared to the P−L− group (experimental control) (Figure 12A). In cells grown on SDA with fluconazole (Figure 12B), it was observed that Apl#1, Apl#4, and Apl#7 in the P+L+ group also were statistically different from the other applications in the analysis, with an increase of 2.29 × 10^2^ copies/µL compared to the P−L− group (Figure 12B).

For the P+L+ group, Apl#7 showed a reduction of *CAP1* gene expression (2.8 × 10^4^ copies/µL) compared to the P−L− group, with the lowest means of the analysis (Figure 12C). In addition, Apl#1, #4, and #7 of the P+L+ group also were statistically different from all other applications. The cells grown with fluconazole (Figure 12D) showed that Apl#7 of the P+L+ group was statistically different from the P−L− group, with a reduction of 0.07 × 10^5^ copies/µL.

For the *ERG11* gene, the results also showed that Apl#7 of the P+L+ group had the lowest expression values of the analysis and was statistically different from the P−L− group, with a reduction of 2.38 × 10^5^ copies/µL (Figure 12E). Furthermore, in the presence of fluconazole, Apl#1, #4, and #7 of the P+L+ group were statistically different from Apl#1, #4, and #7 of the P−L− (experimental control), with 3.2 × 10^3^ copies/µL of expression reduction (Figure 12F).

### 3.4. Results of RT-qPCR for aPDT Mediated by CUR

**Figure 13:**
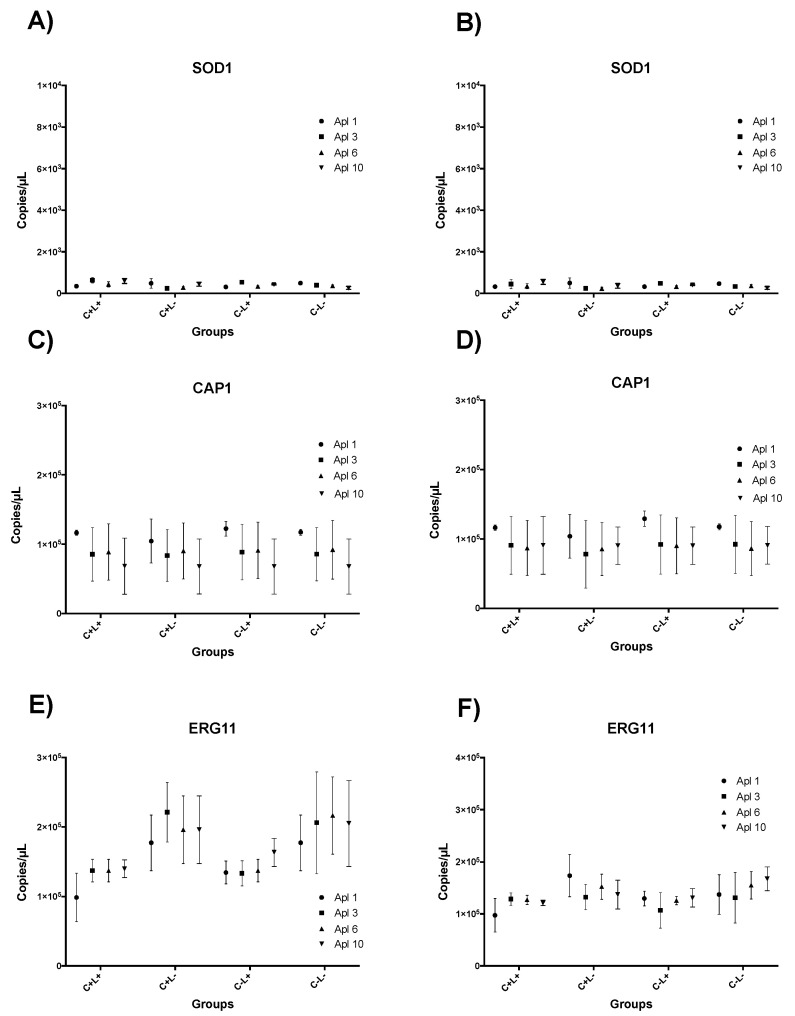
Graphic representation of mean ± confidence interval of 95% of *SOD1* (**A**,**B**), *CAP1* (**C**,**D**), and *ERG11* (**E**,**F**) gene expression in each experimental group of *C. albicans* cells removed from biofilm cultured in SDA without (**A**,**C**,**E**) and with (**B**,**D**,**F**) fluconazole after first (Apl#1), third (Apl#3), sixth (Apl#6) and tenth (Apl #10) application of CUR-mediated aPDT (C+L+), FS control (C+L−), light control (C−L+), and experiment control group (C−L−). Minimum and maximum values of the copies/µL error bar represent the lower and upper limits of the confidence interval, respectively. The non-intersection of error bars denotes statistical difference (*p* < 0.05).

For the *SOD1* and *CAP1* genes, in the CUR-mediated aPDT, no significant difference was observed between the applications (Apl#1, Apl#3, Apl#6, and Apl#10) for all groups evaluated (C+L+, C−L+, C+L−, and C−L−), regardless of the presence or absence of fluconazole (Figure 13A–D).

For the *ERG11* gene (Figure 13E), Apl#1 of the C+L+ group was statistically different from the C−L− group, with a reduction of 2.87 × 10^5^ copies/µL when cells were grown on SDA plates without fluconazole. However, in cells grown on SDA with fluconazole, there was no difference between the applications (Apl#1, Apl#3, Apl#6, and Apl#10) in the C+L+ group or the C−L− group (Figure 13F).

## 4. Discussion

The repeated application of antimicrobial treatment at low (sub-lethal) concentrations can make a microbial population resistant or tolerant [14]. The possible capacity of a microorganism to develop antimicrobial resistance mediated by aPDT has encouraged research in this area [7,15,18,27]. The production of ROS caused by sub-lethal applications of aPDT may not be sufficient to promote the inactivation of a microorganism; however, the accumulation of ROS in the cell can cause the changes responsible for selecting the least susceptible strains (or cell clones) or more tolerant ones that would survive the treatment [11,15]. Nevertheless, there is a lack of knowledge on the effect of successive applications of aPDT at sub-lethal doses on *C. albicans* biofilm. Thus, this study investigated the behavior of *C. albicans* recovered after successive applications of aPDT (mediated by two PS) at sub-lethal doses by evaluating the cell viability, ROS production, and gene expression. As an additional challenge to cells subjected to aPDT, the cultures were plated on SDA with and without FLU to simulate a possible clinical situation in which fluconazole is prescribed [20].

Regarding the viability of planktonic cultures of *C. albicans* submitted to aPDT mediated by PDZ and CUR, the results showed a reduction of 64.2% and 59.8%, respectively, between the first (Apl#1) and third application (Apl#3), when plated on SDA without FLU. In addition, after the fourth application (Apl#4), the presence of viable colonies was not observed. In a previous study, five applications of aPDT were necessary for the number of colonies to decrease to the point of not allowing recovery [7]. This result can be attributed to the methodology based on the recovery and recultivation of survivals cells between the cycles. The present study corroborates the results of a previous study that observed a 3 log_10_ reduction in *C. albicans* in planktonic culture with an equivalent killing efficacy of 99.9% after 20 cycles of aPDT mediated by a phthalocyanine derivative associated with LED (30 J/cm^2^) [16]. The application of 50 and 75 μg/mL of PDZ associated with LED (37.5 J/cm^2^) resulted in the complete inactivation of clinical isolates of *C. albicans* in planktonic cultures [6]. In addition, the results indicated that the sub-lethal dose (25 μg/mL of PDZ associated with 18 J/cm^2^) reduced the viability of *C. albicans* by approximately 1.6 log_10_, a value close to that found in the present study after the first application of PDZ-mediated aPDT (1.4 log_10_ CFU/mL) [6]. Previous studies have reported the complete inactivation of planktonic cultures of *C. albicans* submitted to aPDT associated with CUR (20 μM) and blue LED light (37.5 J/cm^2^–455 nm) [5]. Therefore, aPDT mediated by PDZ and CUR, when applied in a sub-lethal dose, could not develop resistance in planktonic cultures of *C. albicans*. The significant reduction in viability between the first and third applications confirms this hypothesis.

In the planktonic cultures submitted to aPDT mediated by PDZ and CUR that were plated on an agar medium with fluconazole, a complete inactivation of *C. albicans* was observed after Apl#3. These results demonstrate that fluconazole has a synergic effect with aPDT in the planktonic cultures of *C. albicans,* increasing the effect of photodynamic treatment. However, this synergic effect was not detected in a previous study that used miconazole antifungal treatment [28]. This divergence might be justified by the type of antifungals used and treatment performed since the *C. albicans* samples were treated with 25 μg/mL miconazole 2 h before the application of aPDT. In addition, different photosensitizers and strains were tested.

After the first application of aPDT mediated by PDZ in planktonic cultures of *C. albicans*, an increase of 430% in ROS production occurred, while a 683% increase happened after Apl#3. The ROS production after the excitation of chlorine e6 has an intracellular origin, and the PDZ penetrates the cell and accumulates metabolites when excited by light [29]. Thus, the high level of ROS observed after the third application of aPDT was probably due to the accumulation of intracellular metabolites that accentuated the oxidative stress suffered by the fungal cell. This high level of ROS production in planktonic cultures was also noted in CUR-mediated aPDT after the third application (66.7% compared to the first application). Thus, the increases in ROS production corroborate with the CFU/mL reduction, as observed before [11].

The results of the present study also demonstrated that aPDT mediated by PDZ on *C. albicans* biofilms promoted a reduction of 6.2 log_10_ after Apl#3. For *C. albicans* biofilms grown on SDA agar with fluconazole, no viable cells were observed after Apl#7. These results also demonstrate that fluconazole potentiated the aPDT effect in *C. albicans*. The results observed corroborate with the previous study when no growth of cells was observed after Apl#5 [7] and confirm that the *C. albicans* cells in biofilms are more tolerant to aPDT than fungal cells in planktonic cultures [4,5]. The polymeric extracellular matrix (ECM) in *C. albicans* biofilms provides additional protection for the cells once it protects them from oxidative stress, making their inactivation difficult [30]. Studies demonstrate that the resistance conferred to conventional antifungals has been related to the difficulty of penetration of the compound in the multilayers of the biofilm [30]; thus, based on the results obtained in the present study, we can suggest that a similar process may occur with photosensitizers in the biofilm. 

For the *C. albicans* biofilms subjected to PDZ-mediated aPDT, after Apl#7, there was an increase of 87% in ROS production compared to the P−L− group. This production was higher than the first aPDT application. Furthermore, regardless of the number of aPDT applications, this group presented the highest ROS values, only similar to the H_2_O_2_ group. Considering that aPDT is a multi-target therapy, the ROS produced after aPDT application can act on the biofilm matrix and the fungal cell surface [31]. Thereby, we can suggest that the increase in ROS production is associated with the observed decrease in *C. albicans* viability.

In addition, after Apl#7 of aPDT mediated by PDZ in *C. albicans* biofilm, the *SOD1* demonstrated higher expression, and when cultured without fluconazole, Apl# 1, #4, and #7 showed higher expression compared to the other groups. These results agree with studies that report that high levels of *SOD1* protect *C. albicans* cells against free radicals caused by oxidative stress [22,24,25]. Our results corroborate those of a previous study [24] that observed an increase in the gene expression (+1.920 copies/uL) after the fifth application of aPDT mediated by PDZ (200 mg/L) associated with red LED (50 J/cm^2^) in fluconazole-resistant *C. albicans* (ATCC 96901). *SOD1* comprises a class of genes (*SODs*) that act in the process of cellular detoxification, which may explain the increase in the expression of this gene, since when subjected to photodynamic treatment, the cells of *C. albicans* underwent a successive process of accumulation of intracellular ROS [32] from the cycles performed.

For *CAP1* gene expression, there was a significant reduction in Apl#1, #4, and #7 of aPDT mediated by PDZ, and this reduction was higher in Apl#7 in the absence of fluconazole. On the other hand, in the presence of fluconazole, a decrease in *CAP1* gene expression was only observed in Apl#7. These results corroborate those obtained in a previous study [25] in which a reduction in *CAP1* gene expression occurred after a single application of lethal doses of aPDT mediated by PDZ (100 and 200 mg/L) and red LED (37.5 J/cm^2^ or 50 J/cm^2^). In an in vivo study, aPDT mediated by PDZ and red LED promoted a reduction in *CAP1* gene expression (difference of approximately 0.33) in fluconazole-resistant *C. albicans* (ATCC 96901) recovered from mice [24]. *CAP1* is a transcription factor that responds to oxidative stress [33,34], and its reduction makes *C. albicans* cells more vulnerable to the host’s defense mechanisms. Therefore, it is possible to suggest that aPDT can reduce the virulence of the fungus. 

The *ERG11* gene showed significant reduction after Apl#7 of aPDT mediated by PDZ. In the presence of fluconazole, the *ERG11* gene expression was reduced after Apl#1, #4, and #7. These results corroborate a previous study that also noted a reduction in ERG11 expression after aPDT [24]. The *ERG11* gene is correlated to fungal membrane ergosterol biosynthesis and plays a crucial role in the development of hyphae and virulence in *C. albicans* [35]. This result is important for understanding the role of the antifungal fluconazole in the protocol performed. The enzyme C-14-α-lanosterol-demethylase, secreted by *ERG11*, is the binding target of azole antifungals [36]. This binding promotes the inhibition of the ergosterol biosynthetic pathway, which results in an accumulation of toxic sterol intermediates that can alter the stability of the membrane and prevent fungal growth [36,37]. In addition, the alteration in the expression of the *ERG11* gene in yeasts can be considered one of the response mechanisms to azole exposure [38]. Therefore, the presence of fluconazole after aPDT may have been able to interfere with ergosterol biosynthesis, weakening cells that were already compromised by aPDT and resulting in the absence of *ERG11* gene expression. In this context, the results showed that fluconazole potentiated aPDT treatments.

In the aPDT mediated by CUR, a ~1.50 log_10_ reduction of *C. albicans* viability in biofilms was observed after Apl#1 and Apl#2, regardless of the presence of fluconazole. Similar results were found in a previous study that evaluated a combined treatment with a sub-lethal dose of aPDT mediated by CUR with biofilms treated with fluconazole until 48 h [39]. In another study, sub-lethal doses of aPDT mediated by CUR (18 J/cm^2^; 40 μM) promoted an 80% reduction in fungi metabolism (XTT assay) [5].

In contrast to the results observed in biofilms subjected to aPDT mediated by PDZ, when the treatment was performed with CUR, an increase in the fungal viability biofilm of ~1.40 log_10_ CFU/mL occurred after Apl#3 to Apl#10. The presence of ECM may explain the increase in the number of viable cells recovered from the biofilms. Since the PS uptake is hampered, the effect of aPDT mediated by CUR may occur mainly in the ECM, protecting *C. albicans* cells from oxidative damage. Thus, CUR could accumulate mainly outside the *C. albicans* cells into the ECM. This physical barrier provided by the ECM is responsible for preventing oxidative stress from reaching the fungal cells [40]. However, the role of aPDT mediated by CUR should be evaluated in further studies. This phenomenon was not noted in aPDT mediated by PDZ. 

Moreover, successive applications of red LED and blue LED reduced the viability of *C. albicans* in planktonic and biofilm cultures. These results corroborate previous studies showing that LED lights are effective in inactivating microorganisms [17]. It has been suggested that antimicrobial light therapy (>380 nm) can activate endogenous photosensitizing molecules within the cells to produce endogenous ROS [41]. Therefore, since the light application can induce endogenous ROS production, an increase in ROS production was also detected in the groups exposed to light alone. Despite the different mechanisms of action, better microorganism inactivation is expected in aPDT than in antimicrobial light application [42].

Planktonic cultures of *C. albicans* are susceptible to successive applications of sub-lethal doses of aPDT mediated by PDZ and CUR with and without the presence of fluconazole, and these aPDT approaches had a high level of ROS after the last application. In addition, the biofilms were more tolerant than the planktonic cultures to the strategies tested. The biofilm of *C. albicans* was susceptible to successive applications of aPDT mediated by PDZ, showing a high level of ROS production and *SOD1* expression, with a reduction of *CAP1* and *ERG11* expression, regardless of fluconazole. Sub-lethal aPDT mediated by CUR had the potential of turning *C. albicans* cells in biofilms more resistant to the therapy since there was an increase in fungal cell viability after Apl#3. Therefore, under the conditions evaluated here, the response of *C. albicans* in biofilms to aPDT depends on the photosensitizer employed.

## Figures and Tables

**Table 1:** Specific primers for the expression analysis of the *SOD1*, *CAP1*, and *ERG11* genes were acquired and standardized with the ideal concentration for the qPCR test.

Gene	Primers	Optimal Concentration (nM)	Tm (°C) Cycle qPCR
*SOD1*	F—TTG AAC AAG AAT CCG AAT CC R—AGC CAA TGA CAC CAC AAG CAG (Zhu et al., 2011) [26]	400	60
*CAP1*	F—AGT CAA TTC AAT GTT CAA G R—AAT GGT AAT GTC CTC AAG (Alonso et al., 2018) [22]	400	50
*ERG11*	F—CCC CTA TTA ATT TTT TCC CTA ATT AC R—CAC GTT CTT TTC TCA CTT TAA TTT CTT TC (Jordão et al., 2021) [24]	300	58

## Data Availability

Additional data are available on request from the corresponding authors.
